# C2H2 Zinc Finger Proteins GIS2 and ZFP8 Regulate Trichome Development via Hormone Signaling in Arabidopsis

**DOI:** 10.3390/ijms26157265

**Published:** 2025-07-27

**Authors:** Muhammad Umair Yasin, Lili Sun, Chunyan Yang, Bohan Liu, Yinbo Gan

**Affiliations:** 1 Zhejiang Key Laboratory of Crop Germplasm, Department of Agronomy, College of Agriculture and Biotechnology, Zhejiang University, Hangzhou 310058, China; umairyasin@zju.edu.cn (M.U.Y.); 12016013@zju.edu.cn (C.Y.); 2 Department of Biostatistics, Vanderbilt University Medical Center, Vanderbilt University, Nashville, TN 37232, USA; lili.sun@vumc.org; 3 College of Agronomy, Hunan Agricultural University, Changsha 410128, China; liubohan@hunau.edu.cn

**Keywords:** *GIS2*, *ZFP8*, trichome development, hormonal crosstalk, transcriptional regulation, *Arabidopsis thaliana*

## Abstract

Trichomes are specialized epidermal structures that protect plants from environmental stresses, regulated by transcription factors integrating hormonal and environmental cues. This study investigates the roles of two C2H2 zinc finger proteins, *GIS2* and *ZFP8*, in regulating trichome patterning in *Arabidopsis thaliana*. Using dexamethasone-inducible overexpression lines, transcriptomic profiling, and chromatin immunoprecipitation, we identified 142 *GIS2*- and 138 *ZFP8*-associated candidate genes involved in sterol metabolism, senescence, and stress responses. *GIS2* positively and directly regulated the expression of *SQE5*, linked to sterol biosynthesis and drought tolerance, and repressed *SEN1*, a senescence marker associated with abscisic acid and phosphate signaling. *ZFP8* modulated stress-related target genes, including *PR-4* and *SPL15*, with partial functional overlap between *GIS* family members. Spatially, *GIS2* functions in inflorescence trichomes via integrating gibberellin-cytokinin pathways, while *ZFP8* influences leaf trichomes through cytokinin and abscisic acid signal. Gibberellin treatment stabilized GIS2 protein and induced *SQE5* expression, whereas *SEN1* repression was gibberellin-independent. Chromatin immunoprecipitation and DEX-CHX experiment confirmed *GIS2* binding to *SQE5* and *SEN1* promoters at conserved C2H2 motifs. These findings highlight hormone-mediated transcriptional regulation of trichome development by *GIS2* and *ZFP8*, offering mechanistic insight into signal integration. The results provide a foundation for future crop improvement strategies targeting trichome-associated stress resilience.

## 1. Introduction

Trichomes, hair-like epidermal structures on plant aerial surfaces, serve critical ecological roles in mitigating herbivory, ultraviolet (UV) radiation, and water loss [1,2,3]. In economically important crops such as cotton and tobacco, trichomes contribute to fiber production and the synthesis of secondary metabolites [4]. In *Arabidopsis thaliana*, trichome development has emerged as a model system for studying cell fate determination, differentiation, and the integration of hormonal and environmental signals. A core regulatory network centered on the MYB-bHLH-WD40 (MBW) complex—comprising *GLABRA1 (GL1)*, *GLABRA3 (GL3)/ENHANCER OF GLABRA3 (EGL3)*, and *TRANSPARENT TESTA GLABRA1 (TTG1)*—activates trichome-promoting genes such as *GLABRA2 (GL2)* [5,6,7,8]. Trichome patterning is administered by two competing models. The activation-inhibition mechanism involves the MBW complex inducing trichome initiation while stimulating inhibitors such as *TRIPTYCHON (TRY)* and *CAPRICE (CPC)*, creating a self-organizing feedback loop to prevent adjacent cell differentiation [9,10,11]. In contrast, the activator-depletion model proposes that trichome precursors sequester *TTG1*, limiting its availability in neighboring cells [10,12].

Hormonal crosstalk intricately modulates trichome development. Gibberellins (GA) promote initiation by destabilizing DELLA repressors, which otherwise suppress GL1 and GIS family proteins [13,14]. Cytokinins (CK) antagonize GA signaling in a dose-dependent manner, reducing trichome density on mature leaves through mechanisms involving *ARABIDOPSIS RESPONSE REGULATOR (ARR)* genes [15,16], while jasmonic acid (JA) synergizes with GA via JAZ protein degradation, releasing *GL3/EGL3* to activate trichome genes [17]. This crosstalk ensures precise spatiotemporal control of trichome development, enabling adaptation to environmental and developmental cues. However, the integration of hormonal signals (e.g., GA, CK, and JA) with environmental factors remains incompletely resolved, particularly the roles of C2H2 zinc finger proteins like *GIS2* and *ZFP8* in mediating these interactions, highlighting the need for further research into these regulatory intersections.

C2H2 zinc finger proteins (ZFPs) function as key transcriptional regulators in trichome development by integrating hormonal signaling and environmental responses through sequence-specific DNA interactions. Plant C2H2 ZFPs are characterized by conserved cysteine (Cys) and histidine (His) residues that coordinate zinc ions, forming a “finger-like” DNA-binding domain [18,19]. This structural configuration enables sequence-specific interactions, often targeting AG/CT-rich motifs such as AGCTNAC, which are critical for regulating developmental and stress-responsive pathways [20,21,22,23,24]. A hallmark of plant-specific C2H2 ZFPs, such as *GIS2* and *ZFP8*, is the QALGGH pentapeptide motif within their zinc finger loops, which enhances DNA-binding specificity to plant cis-elements and distinguishes them from animal counterparts [20,25,26]. Functionally, these proteins act as molecular hubs, integrating environmental and endogenous signals to regulate diverse processes, including development, hormone signaling, and abiotic stress responses [27]. For example, the C2H2 ZFP *SUPERMAN* maintains floral meristem boundaries by repressing *WUSCHEL* expression [28,29], while *ZAT12* enhances oxidative stress tolerance by activating ROS-scavenging enzymes [30]. Similarly, *STZ/ZAT10* integrates salt and drought responses through ABA-dependent pathways [31]. A subset of C2H2 ZFPs, including *GIS*, *GIS2*, and *ZFP8*, specifically regulate trichome development through hormonal signals. *GIS* promotes trichome initiation on inflorescence stems through gibberellin (GA) signal upstream of *GLABRA1 (GL1)* [32], while *GIS2* and *ZFP8* regulate trichome development by integrating gibberellin (GA) and cytokinin (CK) pathways. *GIS2* and *ZFP8* exhibit distinct spatial roles: *GIS2* primarily influences floral organ trichomes, whereas *ZFP8* modulates trichomes on stems and leaves [13,33]. Despite their conserved QALGGH motif and overlapping regulatory roles, key questions remain unresolved. These include the direct transcriptional targets of *GIS2* and *ZFP8*, their interactions with hormone pathways, and the evolutionary drivers of their functional divergence. Collectively, C2H2 ZFPs exemplify functional versatility, bridging growth regulation and stress adaptation. Their structural conservation, coupled with lineage-specific motifs like QALGGH, highlights their evolutionary specialization in plant gene regulatory networks. Future studies should prioritize elucidating their context-specific DNA-binding dynamics and signaling crosstalk in developmental and stress contexts, as addressed in this work.

Hormonal crosstalk between gibberellin (GA) and cytokinin (CK) signaling pathways represents an essential regulatory axis in trichome regulation, with the *GLABROUS INFLORESCENCE STEMS (GIS)* family of C2H2 zinc finger proteins (ZFPs) acting as key molecular mediators. GA promotes trichome initiation by destabilizing DELLA repressor proteins, which suppress the expression of *GIS* family genes (e.g., *GIS*, *GIS2*, and *ZFP8*) [13,32,34]. Conversely, CK antagonizes GA signaling in a dose-dependent manner, reducing trichome density through mechanisms involving *ARABIDOPSIS RESPONSE REGULATOR (ARR)* genes, though the molecular intermediates linking these pathways remain unidentified [15,16]. Recent studies highlight the hierarchical regulation within the *GIS* family: GA and CK signals converge to regulate trichome density, with *GIS2* and *ZFP8* exhibiting distinct hormonal sensitivities [13,33,35]. Functional redundancy and spatial specialization exist among *GIS* paralogs; *GIS2* primarily regulates floral trichomes, whereas *ZFP8* governs stem and leaf trichomes, partially compensating for *GIS* loss [36].

Despite these advances, critical knowledge gaps persist. The regulatory cascades downstream of *GIS2* and *ZFP8*, including their putative targets such as *PR-4* and *SPL15* (already characterized for *GIS*), remain uncharacterized. Furthermore, the mechanisms by which *GIS2* and *ZFP8* differentially integrate GA and CK signals are unclear, as are the evolutionary drivers underlying their promoter-binding specificity and sub-functionalization. Although *ZFP5* has been identified as a GA-responsive regulator linking *GIS* and *ZFP8* [37,38,39], it is unknown whether *GIS2* and *ZFP8* directly mediate hormone signaling or require additional intermediaries. Additionally, the extent of functional redundancy and divergence among *GIS* family members in fine-tuning trichome development in response to environmental cues remains unresolved. Addressing these questions will clarify how hormonal and developmental signals orchestrate trichome patterning through the *GIS* network, offering broader insights into plant developmental plasticity.

Trichome development, a key determinant of plant and environment interactions, is dynamically regulated by hormonal and environmental cues through specialized transcription factors. C2H2 zinc finger proteins play a crucial role in coordinating growth and stress adaptation; however, the mechanistic basis by which homologs *GIS2* and *ZFP8* integrate gibberellin (GA) and cytokinin (CK) crosstalk to modulate trichome patterning remains unclear. This study addresses this gap by pursuing three objectives: identifying transcriptional targets of *GIS2* and ZFP8, using chromatin immunoprecipitation (*ChIP*) and DEX-CHX methods integrated with transcriptomic profiling, and examining their roles in sterol metabolism (*SQE5*), senescence (*SEN1*), and stress adaptation (*PR-4*, *WRKY25*); investigating how GA and CK signaling influence their activity, through hormone responsiveness assays, in the context of promoter-binding specificity and functional divergence; and comparing potential redundancy and specialization between *GIS2* and ZFP8 by analyzing single and double mutants (*gis2 zfp8*) and RNAi lines, focusing on shared targets like *SPL15* that influence trichome patterning during developmental phase transitions. By synthesizing *ChIP*-qPCR, microarray analysis, and mutant characterization, this work examines how *GIS2* and *ZFP8* may have evolved divergent regulatory networks while retaining overlapping functions in trichome development. The significance of this research lies in its potential to expand the trichome regulatory framework, offering mechanistic insights into how *C2H2* zinc finger proteins balance redundancy and specialization to coordinate development with environmental responses. Notably, *GIS2* regulation of *SQE5* and *SEN1* suggests possible links between trichome morphogenesis and drought or *ABA* signaling, while *ZFP8* regulation of *PR-4* (pathogen defense) may connect trichome traits to biotic resilience. Elucidating *GIS2* and *ZFP8* networks could provide a conceptual foundation for future strategies such as CRISPR-based approaches to engineer crops with optimized trichome features and enhanced adaptability to environmental challenges.

## 2. Results

### 2.1. Expression of GIS2 and ZFP8 in the pOp6/LhGR System

**Figure 1:**
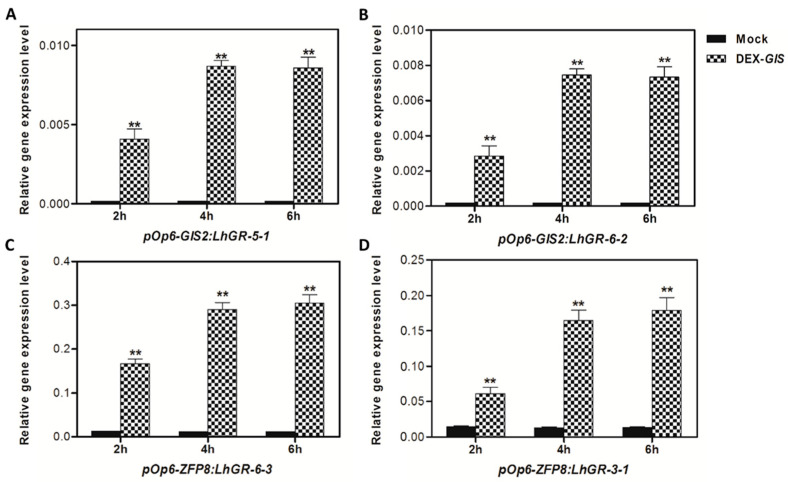
The expression of *GIS2* and *ZFP8* in the main stem after treatment with DEX for 2 h, 4 h, and 6 h. (**A**) The expression of *GIS2* in transgenic pOp6:*GIS2*-5-1 line. (**B**) The expression of *GIS2* in transgenic pOp6:*GIS2*-6-2 line. (**C**) The expression of *ZFP8* in transgenic pOp6-*ZFP8*:LhGR-6-3 line. (**D**) The expression of *ZFP8* in transgenic pOp6-*ZFP8*:LhGR-3-1 line. Note: The relative gene expression value was calculated by using *UBQ10* as the housekeeping gene against the wild type. Error bars represent standard error. The *t*-test was calculated at 1% (*p* < 0.01 with significant level **) probability.

To analyze the temporal expression profiles of *GIS2* and *ZFP8* under dexamethasone (Dex) induction, T2 transgenic lines of *pOp6-GIS2:LhGR* and *pOp6-ZFP8:LhGR* were screened on kanamycin- and hygromycin-containing media. When the primary stems reached approximately 5 cm, Dex was applied, and tissues were harvested at 2 h, 4 h, and 6 h post-treatment. RNA extraction and subsequent cDNA synthesis revealed that *GIS2* expression in lines *pOp6-GIS2:LhGR-5-1* and *pOp6-GIS2:LhGR-6-2* increased progressively and reached a stable level at 4 h after Dex induction (Figure 1A,B), closely resembling the temporal pattern of the related *GIS* gene [40]. Similarly, *ZFP8* expression in lines *pOp6-ZFP8:LhGR-3-1* and *pOp6-ZFP8:LhGR-6-3* exhibited comparable kinetics, with peak transcript levels observed at 4 h (Figure 1C,D). Notably, error bars in Figure 1A–D reflect minimal biological variability (SEM, *n* = 3), and Student’s *t*-test analysis confirmed statistically significant induction (*p* < 0.01) at 4 h and 6 h compared to mock controls (Figure 1 legend). Based on these results, the 4 h time point was selected for downstream microarray analysis. Tissues from the high-expression lines *pOp6-GIS2:LhGR-5-1* and *pOp6-ZFP8:LhGR-6-3* were used to examine Dex-responsive gene expression changes. This unified experimental framework allowed consistent analysis of early transcriptional responses potentially mediated by *GIS2* and *ZFP8*, providing a foundation to identify candidate downstream genes regulated under inducible conditions.

### 2.2. Identification of Downstream Genes Involved in GIS2 and ZFP8 Regulatory Pathways via Microarray Data Analysis

To elucidate the molecular mechanisms by which *GIS2* and *ZFP8* may influence trichome development, transcriptomic profiling was performed using Affymetrix microarrays on *Arabidopsis* wild type (WT), mutants (*gis2*, *zfp8*), dexamethasone (Dex)-induced overexpression lines (*pOp6-GIS2:LhGR-5-1* and *pOp6-ZFP8:LhGR-6-3*), and mock-treated controls, with three biological replicates per condition. In *gis2* mutants, 1603 genes were significantly upregulated (fold-change > 1.5, *q* < 0.01) and 1016 downregulated (fold-change < 0.67, *q* < 0.01) compared to WT, while Dex-induced *GIS2* overexpression led to 641 upregulated and 921 downregulated genes. Cross-comparison identified 58 genes upregulated in *gis2* but repressed upon *GIS2* induction and 84 genes downregulated in *gis2* but induced following Dex treatment, yielding 142 *GIS2*-associated differentially expressed genes. Similarly, *zfp8* mutants exhibited 1214 upregulated and 831 downregulated genes, while *ZFP8* overexpression resulted in 1446 upregulated and 1743 downregulated transcripts. Among these, 85 genes were upregulated in *zfp8* but suppressed by Dex-induced *ZFP8*, and 53 genes were downregulated in *zfp8* but induced upon overexpression, identifying 138 *ZFP8*-associated candidates. Gene ontology (GO) enrichment analysis showed that these gene sets were significantly associated with catalytic activity, metabolic processes, binding, and cellular components. Notably, *GIS2*-associated genes included *At5g24150 (SQE5)*, involved in sterol biosynthesis, and *At4g35770 (SEN1)*, linked to senescence regulation, while *ZFP8*-associated candidates featured *PR-4* (*At3g04720*) and *SPL15 (At3g57920)*, genes related to the stress response and phase transition (Table 1, Table 2, Table 3 and Table 4). These overlapping genes with inverse expression profiles between mutant and overexpression lines are considered as potential transcriptional targets and were further evaluated by using ChIP-qPCR and hormone response assays. Collectively, the data support a model in which *GIS2* and *ZFP8* participate in partially overlapping but distinct regulatory networks influencing trichome development through transcriptional modulation of stress- and hormone-related genes.

### 2.3. Transcription Factor Analysis of 142 GIS2-Regulated Genes and 138 ZFP8-Regulated Genes

**Figure 2:**
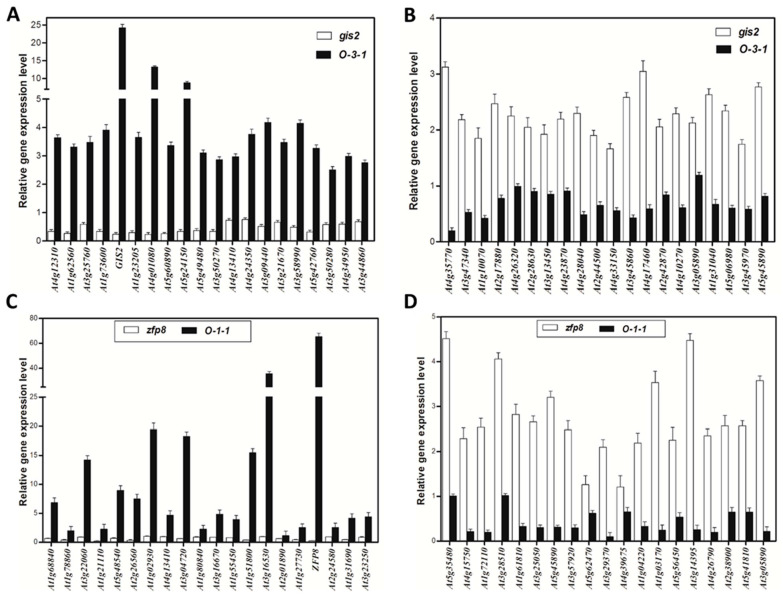
The relative expression of 40 selected genes in the main stem of *gis2*, 35S:*GIS2* (O-3-1), *zfp8*, and 35S:*ZFP8* (O-1-1) compared to the wild type (WT). (**A**) The expression of 20 lowest expressed genes among 142 genes in *gis2*/WT microarray data. (**B**) The expression of 20 most highly expressed genes among 142 genes in *gis2*/WT microarray data. (**C**) The expression of 20 lowest expressed genes among 138 genes in *zfp8*/WT microarray data. (**D**) The expression of 20 most highly expressed genes among 138 genes in *zfp8*/WT microarray data. Note: The relative gene expression value was calculated by using *UBQ10* as the housekeeping gene against the wild type. Error bars represent standard error.

Microarray analysis identified 142 *GIS2*-associated genes, comprising 84 genes downregulated and 58 genes upregulated in the *gis2* mutant compared to the wild type. The top 20 most significantly downregulated genes (Table 1) and the top 20 most upregulated genes (Table 2) were selected for validation via quantitative PCR (q-PCR). In *35S:GIS2* overexpression lines, 19 out of 20 downregulated genes (excluding *GIS2* itself) showed expression patterns that positively correlated with *GIS2* levels, while the 20 upregulated genes displayed inverse trends (Figure 2A,B). Among these, At4g01080 (hypothetical protein) and At5g24150 (*SQE5*, squalene monooxygenase) showed pronounced upregulation in *35S:GIS2* and marked downregulation in *gis2*, suggesting their transcription is influenced by *GIS2* activity. Conversely, *At4g35770* (*SEN1*, senescence-associated gene), *At4g17460* (homeobox-leucine zipper protein), *At3g45860* (receptor-like kinase), and *At5g45890* (cysteine protease) were identified as candidates potentially repressed by *GIS2*. For *ZFP8*, microarray analysis identified 138 associated genes, with 53 downregulated and 85 upregulated in the *zfp8* mutant. q-PCR validation of the 20 most significantly altered genes (Table 3 and Table 4) revealed that 19 out of 20 downregulated genes (excluding *ZFP8*) exhibited a positive correlation with *ZFP8* expression, while the 20 upregulated genes were inversely regulated (Figure 2C,D). Key *ZFP8*-associated targets included *At1g02930* (glutathione S-transferase), *At3g22060* (unknown protein), *At3g04720* (*PR-4*), *At1g51800* (receptor kinase), and *At3g16530* (lectin-like protein), many of which are known to be involved in abiotic and biotic stress responses. Notably, *PR-4* appeared in both *GIS2* and *ZFP8* gene sets (Table 2 and Table 3), highlighting a point of functional convergence within their regulatory networks. These findings suggest that both *GIS2* and *ZFP8* function as transcriptional activators of positively regulated targets and potential repressors of negatively correlated genes, as reflected in the inverse expression profiles validated by q-PCR. The high concordance between microarray data and q-PCR results (Figure 2) supports the robustness of the identified gene sets. The inclusion of stress-associated (*At3g04720* and *At1g02930*) and developmental (*At3g16530*) genes among *ZFP8*-regulated targets further underscores its dual role in trichome patterning and environmental adaptation.

### 2.4. q-PCR Validation of Downstream Genes Regulated by GIS2 and ZFP8

**Figure 3:**
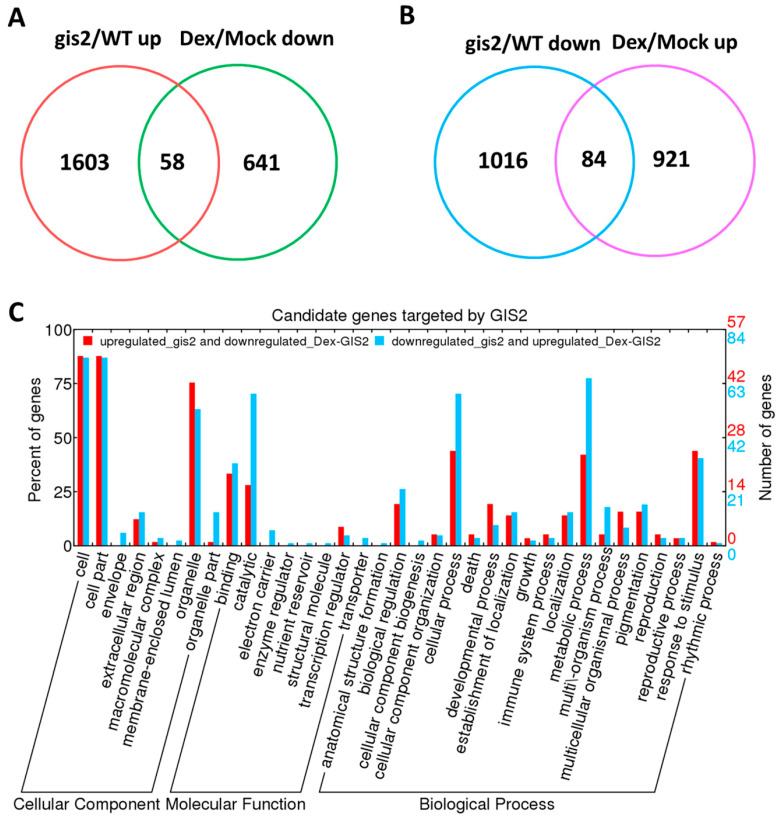
The downstream genes regulated by *GIS2* identified through microarray assay. (**A**) Upregulated genes in *gis2*/WT VS downregulated genes in DEX-induced *GIS2*/mock. (**B**) Downregulated genes in *gis2*/WT VS upregulated genes in DEX-induced *GIS2*/mock. (**C**) Candidate genes regulated by *GIS2* by GO category analyses.

**Figure 4:**
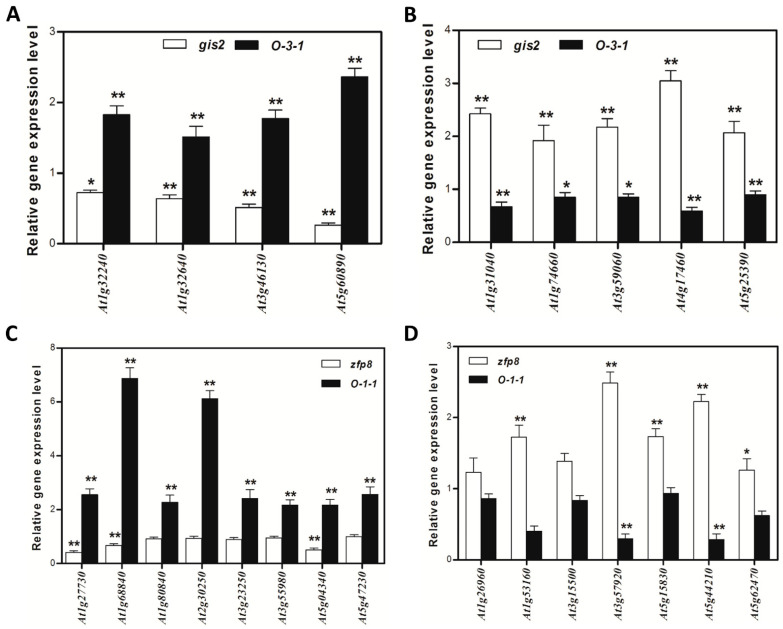
The relative expression of nine selected transcriptional factors in the main stem of *gis2* and 35S:*GIS2* (O-8), and the relative expression of fifteen selected transcriptional factors in the main stem of *zfp8* and 35S:*ZFP8* (O-1-1). (**A**) The relative expression of 4 transcriptional factors positively regulated by *GIS2*. (**B**) The relative expression of 5 transcriptional factors negatively regulated by *GIS2*. (**C**) The relative expression of 8 transcriptional factors positively regulated by *ZFP8*. (**D**) The relative expression of 7 transcriptional factors negatively regulated by *ZFP8*. Note: The relative gene expression value was calculated by using *UBQ10* as the housekeeping gene against the wild type. Error bars represent standard error. The t-test was calculated at either 5% (*p* < 0.05 with significant level *) or at 1% (*p* < 0.01 with significant level **) probability.

**Figure 5:**
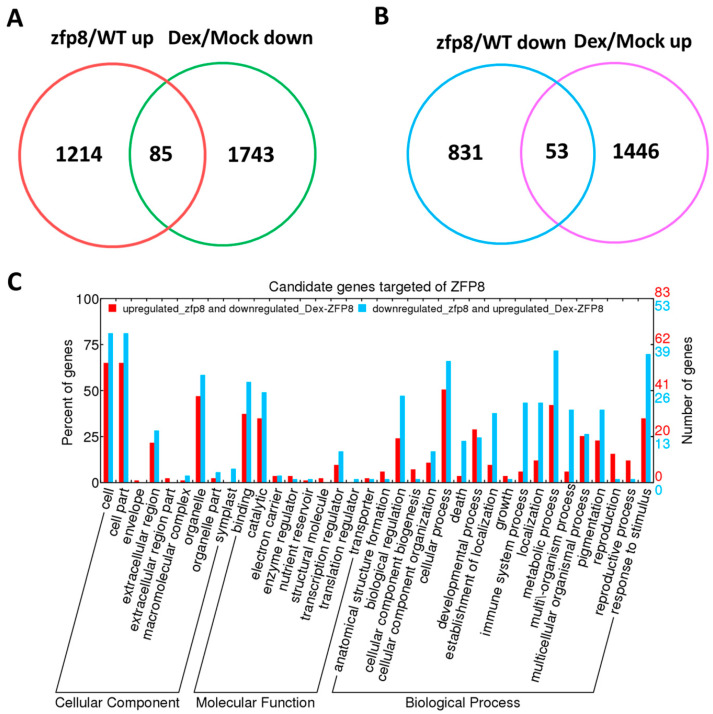
The downstream genes regulated by *ZFP8* identified through microarray assay. (**A**) Upregulated genes in *zfp8*/WT vs. downregulated genes in DEX-induced *ZFP8*/mock. (**B**) Downregulated genes in *zfp8*/WT vs. upregulated genes in DEX-induced *ZFP8*/mock. (**C**) Candidate 138 (85 + 53) genes regulated by *ZFP8* by GO category analyses.

Transcription factors (TFs) play pivotal roles in regulating trichome development. Among the 142 *GIS2*-associated genes (Figure 3), 10 were annotated as transcription factors, including *GIS2* itself. Of these, four TFs appeared to be upregulated and five downregulated in response to *GIS2* activity (Table 5 and Table 6). Quantitative PCR (q-PCR) in *gis2* mutants and *35S:GIS2* (O-3-1) overexpression lines confirmed that the four upregulated TFs displayed ≥ 1.5-fold increased expression in overexpression lines (Figure 4A), with *At5g60890* (an MYB family transcription factor homologous to *ATR1/MYB34*) showing the strongest induction. Among the downregulated TFs, *At4g17460* (homeobox-leucine zipper protein *HAT1*), known to influence trichome initiation, was notably suppressed in *gis2* mutants (Figure 4B). For the 138 *ZFP8*-associated genes (Figure 5), 16 encoded TFs, including *ZFP8*. Of these, eight were upregulated and seven downregulated in the *zfp8* mutant background. q-PCR validation in *zfp8* mutants and *35S:ZFP8* (O-1-1) lines revealed ≥ two-fold induction of the eight positively regulated TFs (Figure 4C), including *AT1G68840* (AP2-EREBP family member *RAV2*) and *AT2G30250* (*WRKY25*), both implicated in stress response signaling. Among the negatively associated TFs, *AT1G53160* (*SPL4*), *AT3G57920* (*SPL15*), *AT5G15830* (a bZIP family TF), and *AT5G44210* (an ERF subfamily member) were significantly upregulated in *zfp8* mutants, suggesting they may be negatively modulated by *ZFP8* (Figure 4D). Importantly, *SPL15* (*AT3G57920*), previously reported as a *GIS* target, was also among the *GIS2*- and *ZFP8*-influenced genes, highlighting a possible regulatory intersection. The consistent negative regulation of *SPL15* and *SPL4* by both *GIS2* and *ZFP8* suggests functional overlap between their pathways, potentially contributing to the coordination of trichome development with phase transition events such as the shift from vegetative to reproductive growth.

### 2.5. Screening of GIS2 Candidate Target Genes Using Dex and Cycloheximide Treatment

**Figure 6:**
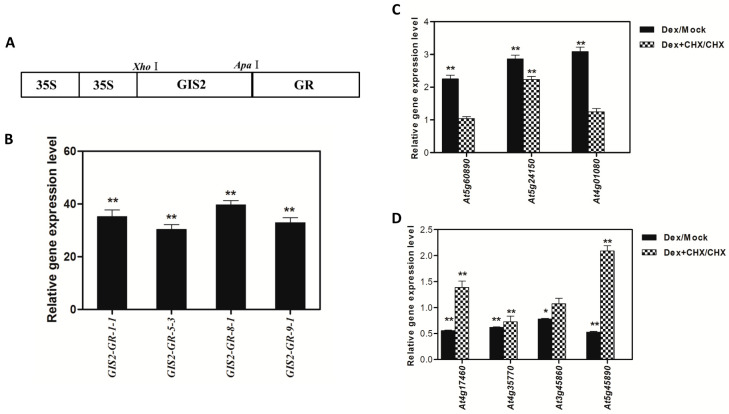
The relative gene expression of *GIS2* in four 35S:*GIS2*-GR::*gis2* transgenic lines compared to the wild type (WT), and the relative gene expression of candidate genes regulated by *GIS2* after treatment with DEX/mock and DEX + CHX/CHX. (**A**) The vector diagram of 35S:*GIS2*-GR. (**B**) The relative gene expression of *GIS2*. (**C**) The relative gene expression of *At5g24150*, *At4g01080*, and *At5g60890* positively regulated by *GIS2*. (**D**) The relative gene expression of *At4g17460*, *At4g35770*, *At3g45860*, and *At5g45890* negatively regulated by *GIS2*. Note: The value was calculated by using *UBQ10* as the housekeeping gene against wild type. Error bars represent standard error. The t-test was calculated at either 5% (*p* < 0.05 with significant level *) or at 1% (*p* < 0.01 with significant level **).

To further assess whether *GIS2* directly regulates the candidate genes identified through transcriptomic profiling, a dexamethasone (Dex)-inducible *35S:GIS2-GR* construct was introduced into *gis2* mutants, resulting in 12 independent transgenic lines (Figure 6A). Among them, line *35S:GIS2-GR::gis2-8-1*, which showed the highest *GIS2* expression by q-PCR (Figure 6B), was selected for analysis. Seedlings were subjected to four treatments: mock, Dex alone, Dex with cycloheximide (CHX), and CHX alone. RNA was extracted 2 h after the second spray for q-PCR analysis. For positively regulated genes including *At5g24150 (SQE5)*, *At4g01080* (hypothetical protein), and *At5g60890 (MYB34)*, expression was significantly induced under Dex treatment compared to mock. Notably, only *SQE5* maintained elevated expression in the Dex + CHX treatment (Figure 6C), suggesting that its regulation by *GIS2* may not require de novo protein synthesis and is, therefore, a strong candidate for direct transcriptional activation. For negatively associated targets, such as *At4g17460 (HAT1)*, *At4g35770 (SEN1)*, *At3g45860* (receptor-like kinase), and *At5g45890 (SAG12)*, Dex treatment reduced transcript levels. Among these, only *SEN1* remained significantly downregulated in the Dex + CHX condition (Figure 6D), implicating it as a putative direct repression target of *GIS2*. These expression trends mirror patterns observed in earlier transcriptomic datasets and are consistent with established protocols [33,41]. While additional assays such as ChIP are needed to conclusively determine direct binding, the CHX inhibition approach provides strong evidence that *SQE5* and *SEN1* are likely direct transcriptional targets of *GIS2*, highlighting their relevance in linking trichome development to hormonal and senescence pathways.

### 2.6. ChIP Analysis Reveals GIS2 Binding to At5g24150 and At4g35770 Promoters

**Figure 7:**
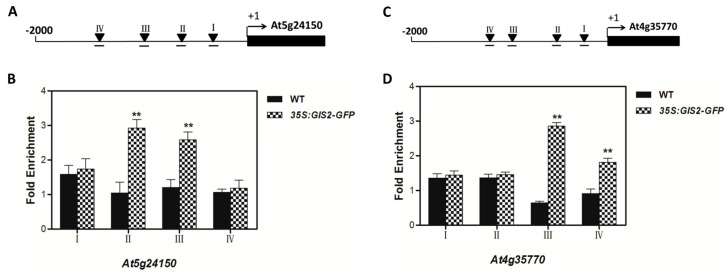
ChIP analysis of *At5g24150* promoter regions bound by *GIS2* and *At4g35770* promoter regions bound by *GIS2*. (**A**) Schematic diagram of the *At5g24150* promoter. The arrowheads indicate the sites containing either a single mismatch or a perfect match from the consensus binding sequence A[AG/CT]CN AC for C2H2 zinc finger proteins. (**B**) Quantitative real-time PCR assay of DNAs after ChIP. (**C**) Schematic diagram of the *At4g35770* promoter. The arrowheads indicate the sites containing either a single mismatch or a perfect match from the consensus binding sequence A[AG/CT]CN AC for C2H2 zinc finger proteins. (**D**) Quantitative real-time PCR assay of DNAs after ChIP. Error bars represent standard error. The t-test was calculated at 1% (*p* < 0.01 with significant level **).

Chromatin immunoprecipitation (ChIP) assays using *35S:GIS2-GFP* transgenic lines provided further evidence for *GIS2* binding to the promoter regions of two candidate target genes, *At5g24150 (SQE5)* and *At4g35770 (SEN1)*, which were previously implicated through transcriptomic and CHX-based expression analyses (Figure 7). Within the 2000 bp upstream regions of both genes, four conserved *C2H2* zinc finger binding motifs (A[AG/CT]CNAC) were identified. ChIP-qPCR revealed that promoter fragments II and III of *SQE5* and fragments III and IV of *SEN1* were significantly enriched following anti-GFP immunoprecipitation (Figure 7B,D), suggesting direct binding by *GIS2*. Functionally, *SQE5* encodes a squalene monooxygenase involved in sterol biosynthesis and has been associated with drought adaptation, similar to its homolog *SQE1* [42]. In contrast, *SEN1* is a senescence-associated gene responsive to darkness and abscisic acid (ABA) signaling, consistent with its known induction in stress-related and developmental transitions [43,44]. These ChIP results corroborate previous q-PCR and expression data (Figure 4 and Figure 6), supporting the model that *GIS2* exerts dual regulatory roles by directly activating positively regulated targets such as *SQE5* and repressing genes like *SEN1*. The promoter-specific binding highlights *GIS2*’s function in modulating both developmental timing and stress responses through targeted transcriptional control (Figure 7A,C).

### 2.7. GA-Induced Expression of GIS2 and Its Downstream Target Genes

**Figure 8:**
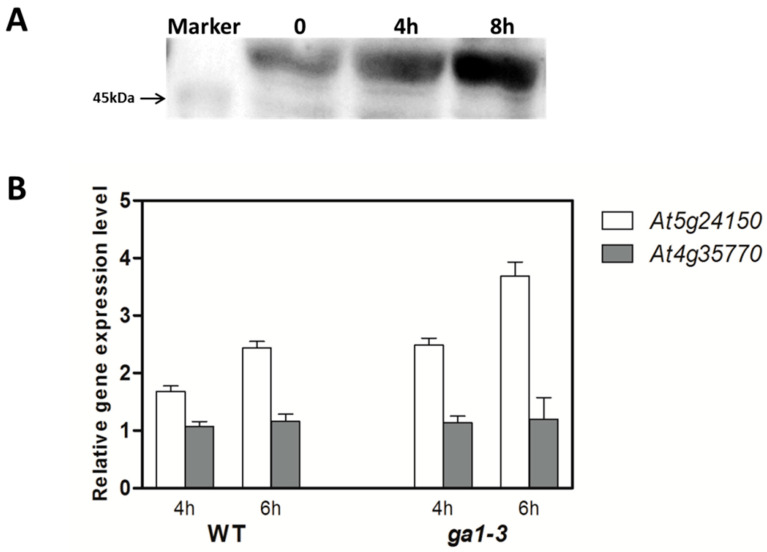
The protein expression of *GIS2* and the gene expression of *At5g24150* and *At4g35770* after treatment with 100 µM *GA*. (**A**) Detection of *GIS2* protein after 4 h and 8 h *GA* (100 µM) treatment by Western blotting. (**B**) The relative gene expression of *At5g24150* and *At4g35770* in inflorescence organs of wild type and *ga1-3* after *GA* (100 µM) treatment for 4 h and 6 h. Note: The value was calculated by using *UBQ10* as the housekeeping gene against the wild type. Error bars represent standard error.

To validate GA-induced regulation of *GIS2* beyond the transcriptional level, we analyzed *GIS2* protein dynamics in *35S:GIS2-GFP* plants treated with 100 µM gibberellic acid (GA). Western blotting revealed a significant increase in *GIS2* protein abundance after 8 h of GA treatment (Figure 8A), confirming GA-dependent regulation at the protein level. This stabilization supports the role of *GIS2* in integrating GA signaling with trichome development, as DELLA protein degradation by GA is known to relieve repression on *GIS2* accumulation [13]. We further examined the GA responsiveness of two *GIS2*-regulated targets: *At5g24150 (SQE5)*, a positively regulated gene, and *At4g35770 (SEN1)*, a negatively regulated one. In wild-type (WT) plants, *SQE5* expression increased significantly after 4 to 6 h of GA treatment (Figure 8B), while *SEN1* exhibited no significant response in either WT or the GA-deficient *ga1-3* mutant (Figure 8B). Given that *SEN1* is known to respond to abscisic acid (ABA) and phosphate starvation [43,44,45], its regulation by *GIS2* may occur through GA-independent pathways or in a context-dependent manner. These results collectively demonstrate that GA enhances *GIS2* protein levels and promotes *SQE5* expression, whereas *SEN1* repression by *GIS2* appears uncoupled from GA signaling. This divergence in hormonal responsiveness highlights distinct regulatory modes—*GIS2* links trichome morphogenesis to sterol biosynthesis via GA-responsive *SQE5*, while modulating *SEN1* through potentially ABA- or nutrient-mediated mechanisms (Figure 8).

### 2.8. Functional Analysis of ZFP8-Regulated Genes in Biological Processes

**Figure 9:**
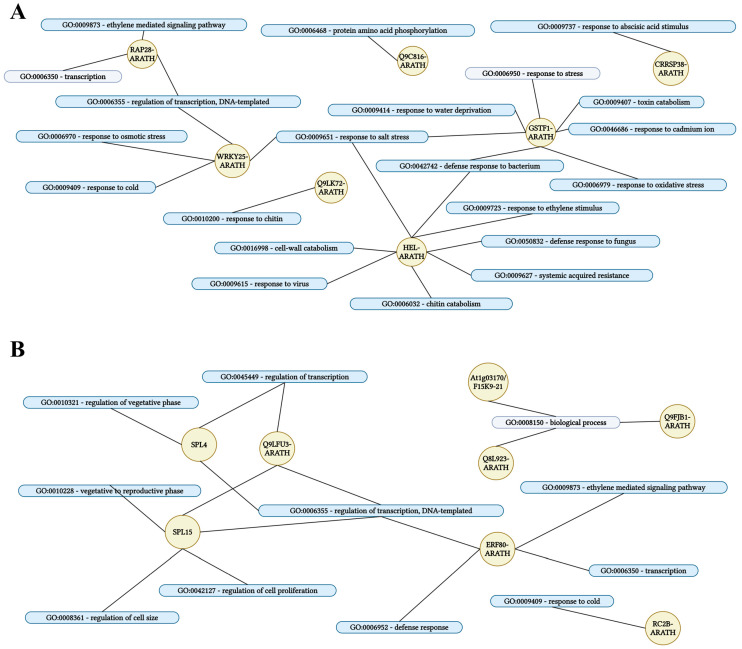
Gene ontology (GO) analysis of significant genes regulated by *ZFP8* involved in biological processes. (**A**) GO analysis of the significant genes upregulated by *ZFP8*. (**B**) GO analysis of the significant genes downregulated by *ZFP8*.

Functional annotation of *ZFP8*-associated genes revealed roles in both stress adaptation and developmental regulation. Upregulated targets such as *At1g02930* (glutathione S-transferase), *At3g22060* (unknown protein), *At3g04720* (*PR-4)*, a pathogenesis-related protein, *At1g51800* (receptor kinase), *At3g16530* (lectin-like protein), and transcription factors *At1g68840* (*RAV2*) and *At2g30250* (*WRKY25*) were enriched in pathways related to hormone signaling (abscisic acid and ethylene), abiotic stress responses (including cold, drought, and salinity), pathogen defense, toxin catabolism, and transcriptional control (Figure 9A). In contrast, downregulated genes such as *At5g35480*, *At3g28510*, *At3g14395*, *At1g03170*, and *At3g05890* and transcription factors *At1g53160* (*SPL4*), *At3g57920* (*SPL15*), *At5g15830* (bZIP-type), and *At5g44210* (ERF family) were linked to ethylene signaling, defense responses, cell expansion, transcriptional repression, and phase transition control (Figure 9B). Although *ZFP8* is a known regulator of trichome patterning on stem leaves [13]—a trait contributing to water retention, temperature buffering, and protection against abiotic stress [2]—the specific roles of its downstream genes in trichome formation remain to be elucidated. Notably, the *ZFP8*-modulated stress-responsive pathways, such as ABA-related signaling via *RAV2* and *WRKY25* and pathogen defense via *PR-4*, align with the protective functions of trichomes, suggesting potential indirect links. The identification of *PR-4* as a shared target of *GIS2* and *ZFP8* (Figure 4, Table 2 and Table 3) supports a role in biotic stress resistance, possibly mediated through trichome architecture. Similarly, *SPL15* (Figure 9B; Table 7 and Table 8), known to influence developmental phase transitions, further connects *ZFP8* activity to growth stage-dependent trichome dynamics. While the functional relevance of these targets in direct trichome development remains unresolved, their integration into hormone and defense pathways underscores *ZFP8*’s broader regulatory capacity. Future studies are necessary to clarify whether these targets contribute directly to trichome morphogenesis or enhance environmental resilience through secondary pathways regulated by *ZFP8*.

## 3. Discussion

Plant tissue and organ morphogenesis is dependent on an exact coordination of cell cycle progression and differentiation, which is mediated by conserved regulators such as cyclin-dependent kinases (CDKs) and cyclins [46]. Metabolic signals, including sugar availability, help to further refine these mechanisms through combining growth with developmental barriers to ensure appropriate cellular patterns [47,48]. Trichome development in *Arabidopsis thaliana* provides a model of this coordination in which epidermal cells move from mitotic division to endoreduplication—a cell cycle variant that increases nuclear DNA content without cytokinesis. This change starts trichome differentiation; trichome size and branching complexity are determined by endoreduplication cycle count, hence defining important features for stress resilience [49]. Recent studies show that, along with nuclear expansion with trichome morphogenesis, histone acetyltransferases like GCN5 and ubiquitin-mediated proteolysis of cell cycle inhibitors dynamically control this process [50,51]. Phytohormones, particularly gibberellins (GAs) and jasmonates (JAs), influence trichome density by connecting developmental cues with stimulation from the environment. GA signaling induces trichome initiation by degrading DELLA repressors via the GID1 receptor, releasing inhibition on trichome-promoting factors [52] (Figure 8A). JA, on the other hand, balances defense priority with growth by altering trichome elongation through MYC transcription factors [53].

The interaction between these pathways provides adaptive plasticity, allowing plants to enhance trichome-mediated defenses in response to biotic or abiotic stress while preserving developmental homeostasis [54]. The C2H2 zinc finger transcription factors GIS, GIS2, and ZFP8, which direct trichome patterning across many plant organs, are fundamental members of this regulatory network. Acting upstream of important regulators like GLABRA1 (GL1), GIS, and GIS2 mostly control trichome initiation on inflorescence stems and branches, integrating GA and cytokinin signals [41]. However, ZFP8 controls trichome development on leaves by mediating cytokinin responses downstream of GL3 and TRY [38]. Our findings extend this paradigm by demonstrating that GIS2 and ZFP8 exhibit bifunctional regulatory roles, directly activating stress-adaptive genes (e.g., *SQE5*; Figure 7A,B, Table 1) while repressing senescence-associated markers (e.g., *SEN1*; Figure 7C,D, Table 2). Spatial specificity is evident: GIS2 predominantly functions in inflorescence stems (Figure 1A,B and Figure 6C,D), where GA stabilizes its protein (Figure 8A) and upregulates *SQE5* (Figure 8B), whereas ZFP8 governs leaf trichomes through cytokinin/ABA pathways (Figure 5C and Figure 9A,B). This divergence mirrors evolutionary sub-functionalization, as seen in cotton homologs, where ZFP8 homologs regulate fiber development [55]. The integration of hormonal cues (e.g., GA-induced GIS2 protein accumulation) and environmental signals (e.g., nutrient stress) suggests that these transcription factors balance developmental programs with stress adaptation. While ChIP-qPCR supports GIS2 binding to *At5g24150* and *At4g35770* promoters, future work will apply EMSA and yeast one-hybrid assays as used in our previous studies to further validate these interactions [41,56].

Transcriptomic analyses and dexamethasone-inducible overexpression systems demonstrate that GIS2 and ZFP8 control trichome development by overlapping but spatially different mechanisms. Microarray profiling of *gis2* and *zfp8* mutants revealed 142 and 138 target genes, respectively (Figure 3 and Figure 5), enhanced in catalytic activity, metabolic control, and stress adaptation—processes fundamental for trichome-mediated abiotic resilience. Notably, GIS2 and ZFP8 exhibit bifunctional regulatory roles, activating subsets of genes (e.g., *SQE5* and *PR-4*) while repressing others (e.g., *SEN1* and *SPL15*) to balance trichome differentiation with broader physiological demands (Figure 2, Figure 3, Figure 4, Figure 5, Figure 6 and Figure 7, Table 1, Table 2, Table 3 and Table 4). For instance, GIS2 directly activates *At5g24150* (*SQE5*), a sterol biosynthesis enzyme critical for drought tolerance (Figure 7A,B), while repressing *At4g35770* (*SEN1*), a senescence marker regulated by ABA and phosphate starvation (Figure 7C,D). This dual functionality positions GIS2 as a molecular integrator linking trichome morphogenesis to stress adaptation—a mechanism conserved in homologs like GIS3, which enhances trichome initiation across tissues [39,41].

ChIP assays confirmed the direct binding of GIS2 to conserved C2H2 motifs in the promoters of *SQE5* and *SEN1* (Figure 7), establishing its role as a transcriptional hub. Similarly, ZFP8 modulates stress-responsive targets such as *PR-4* (pathogenesis-related protein) and *SPL15* (squamosa promoter-binding protein), the latter co-regulated by GIS (Figure 2D and Figure 9B; Table 8). This regulatory overlap suggests functional convergence, particularly in coordinating trichome development with phase transitions. For example, *SPL15* is a mediator of shoot meristem phase transitions [57], which is repressed by both GIS2 and ZFP8 (Figure 4D), implying synchronized timing of trichome patterning and reproductive growth. Recent studies in cotton homologs further underscore the conserved role of ZFP8 in fiber development and photosynthetic efficiency [55], suggesting evolutionary selection for stress-responsive trichome regulation.

The spatial and functional divergence between GIS2 and ZFP8 raises questions about redundancy and crosstalk. Does *SPL15* act as a central hub, synchronizing trichome development and flowering? Do interactions with TOE1/TOE2 transcription factors [39] enable compensatory regulation under stress? Addressing these questions requires comparative analyses of DNA-binding landscapes across tissues and conditions. CRISPR-based editing or single-cell transcriptomics [58] could resolve spatiotemporal dynamics of these networks in crops like cotton, where trichome density correlates with drought tolerance. Furthermore, elucidating the ecological roles of *SQE5* (sterol-mediated drought resilience) and *PR-4* (antifungal defense) could inform strategies to engineer crops with optimized trichome traits. By bridging mechanistic insights from *Arabidopsis* (Figure 1, Figure 2, Figure 3, Figure 4, Figure 5, Figure 6, Figure 7, Figure 8 and Figure 9) with translational applications, this work advances both basic research and agricultural innovation in a changing climate.

The regulatory overlap between ZFP8 and GIS2 is evidenced by their common targets, such as *At3g04720* (*PR-4*) and *At3g57920* (*SPL15*), the latter serving as a crucial integrator of developmental signals at the shoot apical meristem [57]. While ZFP8 primarily directs trichome formation on stem leaves [38], its targets are enriched in ABA signaling and drought defense pathways (Figure 9A), supporting trichome roles in stress mitigation [41]. Conversely, GIS2 links trichome development to sterol metabolism via *SQE5* (Figure 7A,B) and senescence regulation via *SEN1* (Figure 7C,D), suggesting broader roles in growth-phase transitions. This functional divergence is suggested by tissue-enriched expression and regulatory profiles: GIS2 showed elevated expression and downstream activation in inflorescence stems (Figure 1A,B and Figure 6C,D), while ZFP8-associated gene ontology terms and target enrichment were more prominent in leaf-related stress pathways (Figure 5C and Figure 9A,B). However, further studies using tissue-specific reporters or functional assays are needed to confirm precise spatial regulation. ZFP8 regulation of SPL15, a key mediator of phase transitions and flowering (Figure 4D; Table 8), suggests it may coordinate trichome development with reproductive timing [57]. While GIS2 did not appear to directly regulate SPL15, its overlap with ZFP8 in targeting other developmental genes points to potential functional convergence. GA signaling stabilizes GIS2 protein (Figure 8A), which coincides with increased expression of its downstream target SQE5 (Figure 8B), a sterol biosynthesis gene associated with drought adaptation [41]. This suggests that GA may indirectly enhance GIS2-mediated transcriptional regulation. SEN1 repression by GIS2 appears independent of GA signaling (Figure 8B). Given previous reports linking SEN1 to ABA and phosphate starvation responses [43,44], its regulation may involve alternative hormonal or nutrient-responsive pathways, highlighting the potential for context-dependent control. Likewise, ZFP8’s interaction with stress-responsive TFs such as *AT1G68840* (*RAV2*) and *AT2G30250* (*WRKY25*) (Figure 4C,D; Table 7) expands its role beyond trichomes to abiotic adaptation. Recent research shows that these TFs interact with TOE1/TOE2 transcription factors [39] to integrate environmental cues with GA and cytokinin signals, optimizing trichome patterning. This aligns with broader evidence that C2H2 TFs act as nodal points in hormonal crosstalk, balancing developmental precision with stress resilience [59].

The spatial and functional divergence between GIS2 and ZFP8 raises questions about redundancy or crosstalk. Does *SPL15* function as a central hub, synchronizing trichome development and flowering? Do TOE1/TOE2 interactions facilitate compensatory regulation under stress? Addressing these questions necessitates comparative analyses of their binding landscapes across tissues and conditions. Furthermore, exploring the ecological relevance of key target genes such as SQE5, previously associated with drought tolerance [42], and PR-4, known for its role in pathogen defense [59], may provide insights to guide crop engineering strategies. While our study supports their transcriptional regulation by GIS2 and ZFP8, physiological validation under stress conditions remains a goal for future work. These mechanisms can be better understood with the help of new tools made possible by recent developments in single-cell transcriptomics and CRISPR-based spatial profiling [58], which combine basic discoveries with agricultural innovations.

Our study establishes *GIS2* and *ZFP8* as central regulators integrating developmental and environmental cues to modulate trichome patterning. Their bifunctional roles as transcriptional activators and repressors—evidenced by targets such as *SQE5* (sterol biosynthesis and drought adaptation) and *SEN1* (senescence and ABA/phosphate signaling)—position them at the intersection of trichome morphogenesis and stress adaptation. Spatial specificity (inflorescence stems vs. leaves) and target divergence enable functional specialization, with GIS2 coordinating GA-dependent *SQE5* activation and ABA-mediated *SEN1* repression, while ZFP8 regulates stress-responsive pathways like *PR-4* and *SPL15* (Figure 2, Figure 7, Figure 8 and Figure 9). These findings extend the known roles of GIS family proteins by linking trichome development to drought resilience via *SQE5* and pathogen defense via *PR-4*. Future studies leveraging CRISPR-based editing or single-cell transcriptomics could dissect the spatiotemporal dynamics of these networks in crops like cotton or maize, where trichome density correlates with stress tolerance. By resolving how GIS2 and ZFP8 balance redundancy and specialization, this work provides a framework to engineer crops with optimized epidermal traits, addressing yield stability in climate-variable environments.

## 4. Materials and Methods

### 4.1. Plant Materials and Growth Conditions

All experiments used *Arabidopsis thaliana* ecotype Columbia (Col-0). The homozygous *gis2* mutant, *35S:GIS2* overexpression line (O-3-1), *zfp8* mutant, and *35S:ZFP8* overexpression line (O-1-1) were utilized. Seeds were surface-sterilized with 5% (*v*/*v*) sodium hypochlorite for 7 min, rinsed five times with sterile distilled water, and stratified on Murashige and Skoog (MS) medium at 4 °C in darkness for 3 days. Seedlings were grown in a controlled environment chamber (20–22 °C, 16/8 h light/dark cycle, photosynthetic active radiation (PAR) at 90–120 µmol m^−2^ s^−1^, 68–78% humidity). Plants were transferred to soil at the 2–3 true leaf stage. Primary stems (3–5 cm in length) were harvested from 23–25-day-old plants (rosette stage), with three biological replicates (1 g per replicate) per genotype, and immediately frozen in liquid nitrogen. For transgenic selection, MS medium supplemented with 50 mg/L kanamycin (for pH2GW7-pOp6 constructs) or 20 mg/L hygromycin (for pK7WGY2 constructs) was used. Resistant seedlings were transferred to soil, and leaf tissue was collected for genomic DNA extraction one week later. 

### 4.2. Gene Cloning and Vector Construction

Gene cloning utilized the Gateway^®^ system (Invitrogen). *GIS2* and *ZFP8* coding sequences were amplified from cDNA using primers containing SalI/NotI restriction sites:

GIS2: 5′-CGGTCGACATGAAGACTTATGATTTCAT-3′ (forward),
5′-AAGCGGCCGCGAGAGGCGTAGATCCAAAC-3′ (reverse).
ZFP8: 5′-CGGTCGACATGGACGAAACCAACGGAC-3′ (forward),
5′-AAGCGGCCGCGAGAGATGAAGATCGAG-3′ (reverse).

PCR products were cloned into pENTR-1A and recombined into destination vectors pH2GW7-pOp6 (kanamycin resistance), pK7WGY2 (hygromycin resistance), or pK7WGY2-GR (for glucocorticoid receptor fusions) via LR reaction. The *35S:GIS2-GR* construct included a C-terminal GFP tag for ChIP validation and was generated using primers with XhoI/ApaI sites. Constructs were introduced into *Agrobacterium tumefaciens* GV3101 via electroporation and transformed into Arabidopsis via the floral dip method [60]. The *pH2GW7-pOp6-GIS2* and *pH2GW7-pOp6-ZFP8* vectors were transformed into *CaMV35S::LhGR* (4c-S5) and Col-0 plants, respectively [61].

### 4.3. RNA Extraction and Transcriptomic Profiling

Total RNA was isolated from Arabidopsis tissues using TRIzol^®^ reagent (Invitrogen (Waltham, MA, USA)) following the manufacturer’s protocol. RNA integrity was verified via agarose gel electrophoresis, and genomic DNA contamination was removed using DNase I (Thermo Scientific). For quantitative real-time PCR (qRT-PCR), 1.5 µg RNA was reverse-transcribed with M-MLV reverse transcriptase (Promega (Madison, WI, USA)). Diluted cDNA (1:4) was amplified using SYBR^®^ Green PCR Master Mix (Takara (Shiga, Japan)) on a Stratagene Mx3005P thermocycler under the following conditions: 95 °C for 1 min, followed by 40 cycles of 95 °C for 5 sec and 60 °C for 20 sec. *UBQ10* served as the internal control, with three technical replicates per sample. Relative expression levels were calculated using the 2−ΔΔCt method [62].

For transcriptomic analysis, total RNA from primary stems of wild type (WT), *gis2*, *zfp8*, *pOp6:GIS2:LhGR-2-2* (DEX-treated), *pOp6:ZFP8:LhGR-6-3* (DEX-treated), and mock-treated controls (three biological replicates per condition) was hybridized to Affymetrix Arabidopsis ATH1 Genome Arrays. Data were processed with Affymetrix^®^ Microarray Suite 5.0, normalized via the RMA algorithm [63], and analyzed using a fold-change threshold of >1.5 or <0.67 with a Benjamini–Hochberg adjusted *q*-value < 0.01. Hierarchical clustering and heatmaps were generated using MeV.

### 4.4. Dexamethasone-Inducible Expression of GIS2 and ZFP8

DEX induction combined with CHX treatment was used to identify candidate genes potentially regulated directly at the transcriptional level as we describe before [33,41]. For dexamethasone (DEX) induction, transgenic lines (*pH2GW7-pOp6-GIS2* or *pH2GW7-pOp6-ZFP8* in the *CaMV35S::LhGR* background) were selected on MS medium containing 50 mg/L kanamycin and 20 mg/L hygromycin. CHX inhibits de novo protein synthesis, allowing identification of primary transcriptional responses that do not require intermediate protein production. Lines showing high DEX-inducible expression via qRT-PCR (e.g., *pOp6-GIS2:LhGR-5-1* and *pOp6-ZFP8:LhGR-6-3*) were retained. At the 3–5 cm primary stem stage, plants were sprayed with 10 µM DEX (Sigma-Aldrich, St. Louis, MO, USA; now part of Merck, headquartered in Darmstadt, Germany) and 0.015% (*v*/*v*) Silwet L-77, while mock controls received 0.015% Silwet L-77 and 0.033% (*v*/*v*) ethanol. Primary stems (1 g per replicate) were harvested at 2 h, 4 h, and 6 h post-treatment (Figure 1), flash-frozen in liquid nitrogen, and stored at −80 °C.

For *35S:GIS2-GR* transgenic lines in the *gis2* mutant background, DEX and cycloheximide (CHX) treatments were performed. Plants were treated twice with 10 µM DEX, mock (0.015% Silwet L-77), 10 µM DEX + 20 µM CHX (Sigma-Aldrich), or 20 µM CHX, with a 4 h interval between treatments. Samples were collected 2 h after the second spray for RNA extraction and expression analysis (Figure 6C,D).

### 4.5. Gibberellic Acid (GA3) Treatment

*35S:GIS2-GFP* transgenic plants, wild type (Col-0), and *ga1-3* mutants were treated at the primary stem stage (~5 cm in length) by spraying with 100 µM GA_3_ (Sigma-Aldrich) dissolved in 0.02% (*v*/*v*) ethanol and 0.015% Silwet L-77 or mock control (solvent only). Primary stems were harvested at 4 h and 6 h post-treatment (Figure 8B), with three biological replicates (1 g per replicate). Samples were immediately frozen in liquid nitrogen and stored at −80 °C until analysis. For Western blotting, total protein was extracted from GA_3_-treated *35S:GIS2-GFP* plants at 4 h and 8 h post-treatment (Figure 8A).

### 4.6. Chromatin Immunoprecipitation (ChIP) Assay

ChIP assays were performed using two independent protocols. For the first method, chromatin was isolated using the EpiQuik™ Plant ChIP Kit (Epigentek, Farmingdale, NY, USA). Immunoprecipitated DNA was analyzed by ChIP-qPCR with primers targeting four promoter fragments per gene (*At5g24150/SQE5* and *At4g35770/SEN1*) (Supplementary Table S1). For the second protocol, 1–3 g of *35S:GIS2-GFP* stem tissue was vacuum-infiltrated with 1% formaldehyde for 10 min, followed by quenching with 2 M glycine. Fixed tissues were homogenized in extraction buffer (0.4 M sucrose, 10 mM Tris-HCl pH 8.0, 10 mM MgCl_2_, 5 mM β-mercaptoethanol, 0.1 mM PMSF, 1× protease inhibitor cocktail [Roche]). Chromatin was sonicated to 500–800 bp fragments using a Bioruptor^®^ (5 cycles: 15 s ON, 60 s OFF). After pre-clearing with protein A-agarose, supernatants were incubated overnight with 2.5 µL anti-GFP antibody (Abmart, Shanghai, China) or anti-HA antibody (Abmart; negative control). Immune complexes were washed sequentially and eluted in 0.1 M NaHCO_3_, 1% SDS. Crosslinks were reversed at 65 °C overnight, followed by Proteinase K digestion. DNA was purified and analyzed by qPCR using β-Tubulin2 as the internal reference [64]. Enriched promoter fragments (II and III for *At5g24150*; III and IV for *At4g35770*) were validated against input controls (Figure 7B,D). Enrichment values were normalized to input DNA. Anti-HA was used as a negative control antibody to ensure specificity.

### 4.7. Western Blot Analysis

Total protein was extracted by homogenizing 0.1–0.3 g of frozen stem tissue in liquid nitrogen and suspending the powder in 3× volume (*w*/*v*) of extraction buffer (100 mM Tris-Cl pH 8.0, 2% SDS, 5 mM EGTA, 10 mM EDTA, 2% β-mercaptoethanol, 1 mM PMSF, 1× protease inhibitor cocktail [Roche]). Lysates were incubated at 65 °C for 10 min to solubilize proteins, followed by centrifugation at 13,000× *g* for 15 min at 4 °C. Supernatants were collected, and 15 µL of protein per sample was denatured in Laemmli buffer (0.5 M Tris-Cl pH 6.8, 25% glycerol, 10% SDS, 0.5% bromophenol blue) at 95 °C for 4 min. Proteins were separated on 12% SDS-PAGE gels at 60 V for 30 min and then 100 V until the dye front migrated and transferred to nitrocellulose membranes using a semi-dry transfer system (Bio-Rad, Hercules, CA, USA) at 15 V for 50 min. Membranes were blocked overnight at 4 °C in 5% non-fat milk in PBST (1× PBS, 0.1% Tween-20) and probed with anti-GFP primary antibody (Abmart, 1:1000 dilution) for 1 h at room temperature. After four washes with PBST, membranes were incubated with HRP-conjugated secondary antibody (Abmart, 1:5000 dilution) for 1 h. Signals were detected using SuperSignal™ West Dura Extended Duration Substrate (Thermo Scientific, Waltham, MA, USA; part of Thermo Fisher Scientific) and imaged on a ChemiDoc™ XRS+ System (Bio-Rad). β-Tubulin (Abmart, 1:2000) served as a loading control (Figure 8A).

### 4.8. Statistical Analysis

Microarray data were processed using Affymetrix^®^ Microarray Suite 5.0, normalized via the RMA algorithm [63], and analyzed with a fold-change threshold of >1.5 or <0.67 and a Benjamini–Hochberg adjusted *q*-value < 0.01. For qRT-PCR, three biological replicates (independent plant samples) and three technical replicates (per cDNA sample) were analyzed. Relative expression levels were calculated using the 2−ΔΔCt method [62,65], with significance determined by two-tailed Student’s *t*-test (*p* < 0.05). ChIP-qPCR data were normalized to β-Tubulin2 and input controls, with significance assessed via one-way ANOVA followed by Tukey’s post hoc test (*p* < 0.05). Hierarchical clustering and heatmaps were generated in MeV (MultiExperiment Viewer; open-source software originally developed at Dana-Farber Cancer Institute, Boston, MA, USA) using Euclidean distance and average linkage. All statistical analyses were performed in GraphPad Prism 9.0, and data are presented as mean ± SD unless stated otherwise. For Western blot quantification, band intensities were normalized to β-Tubulin using Image Lab™ Software (developed by Bio-Rad Laboratories, Hercules, CA, USA).

## 5. Conclusions

This study explores the dual roles of *GIS2* and *ZFP8* as transcriptional regulators potentially coordinating trichome development with stress adaptation in *Arabidopsis*. Using transcriptomic profiling, ChIP assays, and hormone responsiveness tests, we found evidence that *GIS2* may positively regulate *SQE5*, linking sterol biosynthesis to drought tolerance, and negatively influence *SEN1*, potentially delaying senescence via *ABA* signaling. Conversely, *ZFP8* was associated with the regulation of stress-responsive targets like *PR-4*, suggesting a role in connecting trichome function to pathogen defense. Spatial specificity with *GIS2* active in inflorescence stems and *ZFP8* in leaves indicates functional divergence, while shared targets such as *SPL15* suggest possible evolutionary conservation. *GA* was observed to stabilize *GIS2* and coincide with *SQE5* induction, whereas *SEN1* repression appeared to occur independently of *GA*, indicating context-dependent regulatory mechanisms. These findings contribute to the understanding of C2H2 zinc finger proteins as transcriptional integrators balancing developmental precision and environmental responsiveness. By clarifying promoter-specific DNA associations and hormone-linked expression trends, this work offers a foundation for future strategies to engineer crops with improved stress resilience and integrates fundamental plant biology with applied agricultural research.

## Figures and Tables

**Table 1:** The 20 lowest expressed genes among 142 genes in *gis2*/WT microarray data.

Gene ID	Description of Genes	gis2/WT	Dex/Mock
At4g12310	Flavonoid 3,5-hydroxylase -like protein	0.26	1.73
At1g62560	Similar to flavin-containing monooxygenase	0.27	1.60
At3g25760	Hypothetical protein	0.27	2.60
At1g73600	Phosphoethanolamine N-methyltransferase	0.28	1.62
At5g06650 *	Zinc finger-like protein	0.29	1.58
At1g23205	Unknown protein	0.31	2.87
At4g01080	Hypothetical protein	0.31	2.31
At5g60890	Myb transcription factor homolog (ATR1)	0.33	1.65
At5g24150	Squalene monooxygenase	0.34	3.28
At5g49480	NaCl-inducible Ca^2+^-binding protein-like	0.35	2.70
At3g50270	Anthranilate N-hydroxycinnamoyl/benzoyltransferase	0.35	1.74
At4g13410	Putative protein Cyclic beta-1-3-glucan synthase	0.35	2.05
At4g24350	Putative protein storage protein	0.37	1.74
At3g09440	Heat-shock protein (At-hsc70-3)	0.37	1.74
At3g21670	Nitrate transporter	0.37	1.79
At3g58990	3-isopropylmalate dehydratase-like protein	0.38	1.50
At5g42760	Putative protein similar to unknown protein	0.38	1.55
At3g50280	Anthranilate N-hydroxycinnamoyl/benzoyltransferase	0.40	1.55
At4g34950	Putative protein	0.41	1.55
At3g44860	AtPP -like protein	0.41	3.03

The “*” represents *GIS2* gene.

**Table 2:** The 20 most highly expressed genes among 142 genes in *gis2*/WT microarray data.

Gene ID	Description of Genes	gis2/WT	Dex/Mock
At4g35770	Senescence-associated protein	30.62	0.40
At3g47340	Glutamine-dependent asparagine synthetase	9.73	0.51
At1g10070	Similar to branched-chain amino acid aminotransferase	6.68	0.60
At2g17880	Putative DnaJ protein	5.57	0.53
At4g26320	Putative protein	4.93	0.62
At2g28630	Putative fatty acid elongase	3.32	0.57
At3g13450	Branched-chain alpha-keto acid dehydrogenase	3.26	0.49
At4g23870	Putative protein predicted proteins	3.22	0.44
At4g28040	Medicago nodulin N21-like protein MtN21 gene	3.22	0.61
At2g44500	Similar to axi 1 protein from Nicotiana tabacum	2.82	0.65
At4g33150	Lysine-ketoglutarate reductase/saccharopine	2.81	0.60
At3g45860	Protein kinase—like receptor-like protein kinase RLK3	2.79	0.44
At4g17460	Homeobox-leucine zipper protein HAT1 (hd-zip protein 1)	2.76	0.52
At2g42870	Unknown protein	2.51	0.52
At4g10270	Probable wound-induced protein wound-induced protein	2.50	0.43
At3g05890	Low-temperature and salt-responsive protein	2.46	0.46
At1g31040	Hypothetical protein predicted by gene finder	2.45	0.60
At5g06980	Unknown protein	2.39	0.44
At3g45970	Putative protein cim1 induced allergen	2.30	0.54
At5g45890	Senescence-specific cysteine protease SAG12	2.08	0.11

**Table 3:** The 20 lowest expressed genes of 138 genes in *zfp8*/WT microarray data.

Gene ID	Description of Genes	zfp8/WT	Dex/Mock
At1g68840	Putative DNA-binding protein (RAV2-like)	0.24	1.75
At1g78860	Hypothetical protein predicted	0.44	1.89
At3g22060	Unknown protein	0.44	3.58
At1g21110	O-methyltransferase	0.47	1.79
At5g48540	33 kDa secretory protein-like	0.50	4.12
At2g26560	Similar to latex allergen from Hevea brasiliensis	0.50	1.73
At1g02930	Glutathione S-transferase	0.52	1.85
At4g13410	Putative protein Cyclic beta-1-3-glucan synthase	0.54	1.60
At3g04720	Similar to wound-induced protein (WIN2) precursor	0.54	1.70
At1g80840	Putative similar to WRKY transcription factor	0.55	1.83
At3g16670	Unknown protein	0.56	1.52
At1g55450	Similar to embryo-abundant protein	0.57	1.55
At1g51800	Receptor protein kinase	0.59	1.88
At3g16530	Putative lectin similar to lectin	0.59	2.37
At2g01890	Putative purple acid phosphatase	0.59	1.51
At1g27730	Salt-tolerance zinc finger protein	0.59	2.16
At2g41940 *	C2H2-type zinc finger protein	0.60	24.21
At2g24580	Putative sarcosine oxidase	0.60	1.56
At1g31690	Putative similar to copper amine oxidase	0.61	2.65
At3g23250	MYB-related transcription factor	0.61	1.66

The “*” represents *ZFP8* gene.

**Table 4:** The 20 most highly expressed genes of 138 genes in *zfp8*/WT microarray data.

Gene ID	Description of Genes	zfp8/WT	Dex/Mock
At5g35480	Unknown protein	3.91	0.36
At4g15750	Hypothetical protein	2.57	0.38
At1g72110	Hypothetical protein	2.55	0.60
At3g28510	Putative mitochondrial protein	2.27	0.61
At1g61810	Putative similar to beta-glucosidase	2.16	0.47
At3g25050	Endoxyloglucan transferase	2.15	0.55
At5g45890	Senescence-specific cysteine protease SAG12	2.04	0.27
At3g57920	Squamosa promoter-binding protein	1.97	0.61
At5g62470	MYB96 transcription factor-like protein	1.92	0.63
At5g30426	Putative protein	1.85	0.61
At3g29370	Expressed protein	1.85	0.42
At4g39675	Expressed protein	1.82	0.42
At1g04220	Putative beta-ketoacyl-CoA synthase	1.82	0.64
At1g03170	Hypothetical protein predicted by gene finder	1.80	0.65
At5g56450	ADP/ATP translocase-like protein	1.79	0.62
At3g14395	Expressed protein	1.76	0.56
At4g26790	Putative APG protein proline-rich protein APG	1.71	0.49
At2g38900	Putative protease inhibitor	1.71	0.51
At5g41810	Unknown protein	1.71	0.63
At3g05890	Low-temperature and salt-responsive protein (LTI6B)	1.70	0.54

**Table 5:** Predicted transcriptional factors among 142 candidate genes positively regulated by *GIS2*.

Gene ID	Transcription Factor	Description of Genes
At5g06650 *	C2H2	Zinc finger (C2H2 type) family protein
AT1G32240	GARP-G2-like	MYB family transcription factor (KAN2)
AT1G32640	bHLH	Basic helix-loop-helix (bHLH) protein (RAP-1)
AT3G46130	MYB	MYB family transcription factor (MYB48)
AT5G60890	MYB	Receptor-like protein kinase (ATR1) (MYB34)

The “*” represents GIS2 gene.

**Table 6:** Predicted transcriptional factors among 142 candidate genes negatively regulated by *GIS2*.

Gene ID	Transcription Factor	Description of Genes
AT1G31040	PLATZ	Zinc-binding protein-related
AT1G74660	ZF-HD	Zinc finger homeobox family protein
AT3G59060	bHLH	Basic helix-loop-helix (bHLH) family protein
AT4G17460	HB	Homeobox-leucine zipper protein 1 (HAT1)
AT5G25390	AP2-EREBP	Encodes a member of the ERF (ethylene response factor) subfamily B-6 of ERF/AP2 family.

**Table 7:** Predicted transcriptional factors among 138 candidate genes positively regulated by *ZFP8*.

Gene ID	Transcription Factor	Description of Genes
*AT1G27730*	C2H2	Identical to salt-tolerance zinc finger protein (ZAT10)
*AT1G68840*	AP2-EREBP	DNA-binding protein RAV2 (RAV2)/AP2
*AT1G80840*	WRKY	Similar to WRKY transcription factor
*AT2G30250*	WRKY	WRKY family transcription factor(WRKY25)
*AT3G23250*	MYB	MYB family transcription factor (MYB15)
*AT3G55980*	C3H	Zinc finger (CCCH-type) family protein
AT2G41940 *	C2H2	Zinc finger (C2H2 type) family protein
*AT5G04340*	C2H2	Zinc finger (C2H2 type) family protein
*AT5G47230*	AP2-EREBP	Encodes a member of the ERF (ethylene response factor)

The “*” represents ZFP8 gene.

**Table 8:** Predicted transcriptional factors among 138 candidate genes negatively regulated by *ZFP8*.

Gene ID	Transcription Factor	Description of Genes
AT1G26960	HB	Homeobox-leucine zipper protein
AT1G53160	SBP	Squamosa promoter-binding protein-like 4 (SPL4)
AT3G15500	NAC	No apical meristem (NAM) family protein (NAC3)
AT3G57920	SBP	Similar to squamosa promoter binding protein-like 9
AT5G15830	bZIP	Similar to common plant regulatory factor
AT5G44210	AP2-EREBP	Encodes a member of the ERF (ethylene response factor) subfamily
AT5G62470	MYB	MYB family transcription factor (MYB96)

## Data Availability

The data presented in this study are available on request from the corresponding author.

## Supplementary Materials

The following supporting information can be downloaded at: https://www.mdpi.com/article/10.3390/ijms26157265/s1.

## Abbreviations

The following abbreviations are used in this manuscript:

ABA	Abscisic Acid
CHX	Cycloheximide
ChIP	Chromatin Immunoprecipitation
CK	Cytokinin
DEX	Dexamethasone
GA	Gibberellic Acid
GFP	Green Fluorescent Protein
qRT-PCR	Quantitative Real-Time Polymerase Chain Reaction
SQE5	Squalene Monooxygenase 5
SEN1	Senescence-Associated Gene 1
TF	Transcription Factor
