# Supplementary material for: C2H2 Zinc Finger Proteins GIS2 and ZFP8 Regulate Trichome Development via Hormone Signaling in Arabidopsis
Source: Int J Mol Sci. 2025 Jul 27;26(15):7265. doi: 10.3390/ijms26157265 (PMC12346838; doi:10.3390/ijms26157265)
Supplement: Supplementary file 1 [file ijms-26-07265-s001.zip › ijms-3724314-supplementary.pdf]

Table S1. Primer pairs used for ChIP assays.

**Gene**	**Sequence（5'-3'）**	**ChIP fragment**
At5g24150 LP1	TTTCTGTTGTCCACATATTTCGTT	ChIP fragment I
At5g24150 RP1	TGAGTGGTGCCTTTTACTCG	ChIP fragment I
At5g24150 LP2	TTTGTTTCAGTTCCGCAAAA	ChIP fragment II
At5g24150 RP2	GGACCAGATAATCCTTTTGAAGC	ChIP fragment II
At5g24150 LP3	ACAAGAGTTTGGCATTGCAG	ChIP fragment III
At5g24150 RP3	GGCTAGCCATGTGGTTGTCT	ChIP fragment III
At5g24150 LP4	TGAACCCGTAAATAACGTTTCTC	ChIP fragment IV
At5g24150 RP4	TTCCCACCTTTAGTGATATTTGG	ChIP fragment IV
At4g35770 LP1	TGCACATGCCTTGAGTATCTT	ChIP fragment I
At4g35770 RP1	GAAGAGAAGCGGCAAAAGAA	ChIP fragment I
At4g35770 LP2	GGGACTCCCCAAAGAAAACT	ChIP fragment II
At4g35770 RP2	TGCCTCCTTGACTGCTTCTT	ChIP fragment II
At4g35770 LP3	GAAGCAGTCAAGGAGGCAAC	ChIP fragment III
At4g35770 RP3	CCATGCATGTGGTAGGATGT	ChIP fragment III
At4g35770 LP4	GGAAGCCGAAATGAAACTGA	ChIP fragment IV
At4g35770 RP4	GCGGAGGAGAGAAGATGATG	ChIP fragment IV
TUB2-LP	TAAATTCTGAACCCATTGTTTCTCA	Control
TUB2-RP	AGTCCGATGATTGGCTTTATTATTC	Control
